# Kinetics, equilibrium, and thermodynamics investigation on the adsorption of lead(II) by coal-based activated carbon

**DOI:** 10.1186/s40064-016-2839-4

**Published:** 2016-07-22

**Authors:** Zhengji Yi, Jun Yao, Mijia Zhu, Huilun Chen, Fei Wang, Xing Liu

**Affiliations:** Key Laboratory of Functional Organometallic Materials of College of Hunan Province, Department of Chemistry and Material Science, Hengyang Normal University, Hengyang, 421008 People’s Republic of China; School of Civil and Environmental Engineering, and National International Cooperation Base on Environment and Energy, University of Science and Technology Beijing, Xueyuan Road No. 30, Haidian District, Beijing, 100083 People’s Republic of China; School of Water Resource and Environmental Engineering, Sino-Hungarian Joint Laboratory of Environmental Science and Health, Chinese University of Geosciences (Beijing), Beijing, 100083 People’s Republic of China

**Keywords:** Adsorption, Coal-based activated carbon (CBAC), Isotherm, Kinetics, Pb(II), Thermodynamics

## Abstract

The goal of this research is to investigate the feasibility of using activated coal-based activated carbon (CBAC) to adsorb Pb(II) from aqueous solutions through batch tests. Effects of contact time, pH, temperature and initial Pb(II) concentration on the Pb(II) adsorption were examined. The Pb(II) adsorption is strongly dependent on pH, but insensitive to temperature. The best pH for Pb(II) removal is in the range of 5.0–5.5 with more than 90 % of Pb(II) removed. The equilibrium time was found to be 60 min and the adsorption data followed the pseudo-second-order kinetics. Isotherm data followed Langmuir isotherm model with a maximum adsorption capacity of 162.33 mg/g. The adsorption was exothermic and spontaneous in nature. The Fourier transform infrared spectroscopy and scanning electron microscopy analysis suggested that CBAC possessed a porous structure and was rich in carboxyl and hydroxyl groups on its surface, which might play a major role in Pb(II) adsorption. These findings indicated that CBAC has great potential as an alternative adsorbent for Pb(II) removal.

## Background

Lead (Pb) is a naturally occurring poisonous metal found in the Earth’s crust. Because of its softness, high malleability, ductility, low melting point and resistance to corrosion, it is widely used in the production of lead acid batteries, alloys, solder, pigments, cable sheathing, rust inhibitors, glazes, ammunition, and plastic stabilizers (Deng et al. 2007). However, its widespread application has led to extensive environmental contamination, human exposure and remarkable public health problems. When lead is accidentally inhaled or ingested into the body, it causes serious diseases and permanent damages to human health (ATSDR 2013). Infants and children are very sensitive to the poisonous effects of lead and can suffer far-reaching and permanent unfavorable health effects, particularly influencing the development of the brain and nervous system. Lead also leads to long-term jeopardy in adults like hypertension, dental damage and impaired renal function. Pregnant women exposed to high levels of lead can cause abortion, stillbirth, premature delivery and congenital malformation (Sedighi et al. 2012). No safe blood level has been determined and all sources of lead exposure to children should be eliminated and avoided. Lead concentrations in drinking water should be at least below the current United States Environmental Protection Agency’s threshold level of 0.05 mg/L (US EPA 2011). Therefore, removal of lead from wastewater is very important to protect public health. Chemical precipitation, ion exchange, solvent extraction, phytoextraction, ultrafiltration, reverse osmosis and electrodialysis are regarded as the traditional methods for elimination of lead ions from aqueous solutions.

In recent years, uptake of Pb(II) by various low-cost adsorbents has become the major focus of numerous investigations. A variety of adsorbents, such as agricultural and forestry residue (Singh et al. 2014), mineral material (Unuabonah et al. 2008), microbial biomass (Feng et al. 2013), and ion exchange resins (Demirbas et al. 2005) have been employed to remove Pb(II) from wastewater. It should be noted that adsorption is so far the most commonly used technology for the removal of toxic metals from wastewater. Activated carbon is a black powdery substance with well-developed porosity, huge internal specific area and relatively high mechanical strength, thereby making it possible to use it as a good adsorption material for wastewater treatments. Heretofore, much work has been done on the adsorptive removal of Pb(II) by using activated carbon materials derived from different sources (Wilson et al. 2006; Mohammadi et al. 2010; Wang et al. 2010; Huang et al. 2014).

However, little investigation has been conducted on the Pb(II) removal by using coal-based activated carbon (CBAC), which is made from anthracite as raw materials. Compared with other carbonaceous adsorbents, CBAC has a variety of advantages, such as structural stability, high mechanical strength, good wear resistance, low adsorption energy and easy regeneration. Therefore, the goal of this research was to examine the potential to apply CBAC to remove Pb(II) from aqueous solutions. Batch experiments were carried out to evaluate the effects of various operation parameters (time, pH, temperature and initial metal concentration) on the Pb(II) adsorption, and some kinetics and isotherm models were used to describe the adsorption process.

## Methods

### Chemical reagents and adsorbent

The CBAC powder was purchased from Guoqing Water Purification Material Co. Ltd. in China and employed as a sorbent for the following lead adsorption experiments. According to the National Standard of China for Activated Nutshell Carbon. Testing, the CBAC pore structure and pore size distribution were determined by ASAP 2020 (Micromeritics). The t-plot and Barrett–Joyner–Halenda (BJH) methods were used to calculate the microporosity and the mesoporosity of CBAC, respectively. Boehm titration method was applied in the characterization of the surface functional groups of CBAC (Boehm 1966). FT-IR spectroscopy was used to detect vibration frequency change in the CBAC. The spectra were collected by a NICOLET iS10 (Thermo Scientific) within the range 500–4000 cm^−1^ using a KBr window. The structural morphology of CBAC surface was characterized via SEM (Model S-4800 Hitachi, Tokyo, Japan) observation.

The Pb(II) stock solution (1000 mg/L) was obtained by dissolving lead nitrate in distilled water. This stock solution was then diluted to those required concentrations and their pHs were adjusted to desired values with 0.1 or 1.0 mol/L of NaOH or HCl solution. All chemicals in this research were of analytical grade and were used as received without any further treatment.

### Batch adsorption procedure

Batch adsorption experiments were carried out in a series of 250 mL Erlenmeyer flask to explore the effects of the aforementioned process variables on Pb(II) removal. Preliminary experiments were also performed to make certain the minimum and maximum levels of each variable. In general, about 100 mL of Pb(II) solution was mixed with a known amount of CBAC powder. Thereafter, the flasks were agitated at 140 rpm on a thermo controlled rotary shaker. Finally, the equilibrated solutions were withdrawn and the adsorbent was separated from them via centrifugation. The residual Pb(II) concentration in the solution was quantified through a standard microtitration method proposed by Li et al. (2002). All the experiments were repeated twice or thrice to confirm the results and the average values are recorded.

The Pb(II) removal efficiency and adsorption capacity of CBAC powder were calculated by using the following equations:

1 $$Ad\% = \frac{{C_{0} - C_{t} }}{{C_{0} }} \times 100$$

2 $$Q_{t} = \frac{{\left( {C_{0} - C_{t} } \right) \times V}}{W}$$

3 $$Q_{e} = \frac{{\left( {C_{0} - C_{e} } \right) \times V}}{W}$$

where *Ad* % is the Pb(II) removal efficiency; *Q*_*e*_ and *Q*_*t*_ are the adsorption capacity (mg/g) at equilibrium and at time *t* (min), respectively; *C*_0_, *C*_*t*_ and *C*_*e*_ are the initial Pb(II) concentration, liquid-phase Pb(II) concentration at time *t*, and equilibrium Pb(II) concentration (mg/L), respectively; *V* is the volume of the aqueous solution (L); *W* is the mass of the adsorbent (g).

### Adsorption kinetics models

Pseudo-first-order and pseudo-second-order kinetics models are usually adopted in kinetics investigations. The pseudo-first-order equation is a simple kinetics model describing the kinetics process of liquid–solid phase adsorption which was put forward by Lagergren (1898). Its nonlinear formula is given as follows:

4 $$Q_{t} = Q_{e} (1 - e^{{ - k_{1} t}} )$$

where *k*_1_ is the rate constant of the pseudo-first-order sorption (min^−1^). Obviously, *Q*_*e*_ and *k*_1_ can be figured out by plotting *Q*_*t*_ versus *t* and by further nonlinear regression analysis.

The pseudo-second-order model based on the adsorption equilibrium capacity may be expressed as the following linear form (Ho and McKay 1999):

5 $$\frac{t}{{Q_{t} }} = \frac{t}{{Q_{e} }} + \frac{1}{{k_{2} Q_{e}^{2} }}$$

where *k*_2_ is the rate constant of pseudo-second-order adsorption [g/(mg·min)]. Obviously, *Q*_*e*_ and *k*_2_ can be determined experimentally by plotting t/*Q*_t_ versus t.

### Adsorption isotherm models

Langmuir and Freundlich equations are commonly adopted to describe the adsorption isotherms. Langmuir model assumes adsorption homogeneity, such as uniformly energetic adsorption sites, monolayer surface coverage, and no interactions between adsorbate molecules on adjacent sites (Langmuir 1918). Freundlich isotherm is applicable to nonideal sorption onto heterogeneous surfaces involving multilayer adsorption (Li et al. 2012). In this study, the Langmuir and Freundlich adsorption equations were both used to correlate the obtained isotherm data.

The linearized Langmuir equation can be expressed as follows:

6 $$\frac{{C_{e} }}{{Q_{e} }} = \frac{1}{{Q_{\hbox{max} } }}C_{e} + \frac{1}{{bQ_{\hbox{max} } }}$$

where *Q*_max_ represents the maximum monolayer adsorption capacity (mg/g), and *b* represents the Langmuir adsorption constant which is related to the adsorption bonding energy (L/mg).

Based on further analysis of the Langmuir equation, the Langmuir adsorption isotherm can be described using an equilibrium parameter (*R*_L_) calculated by the following equation (Szlachta and Wojtowicz 2013):

7 $$R_{L} = \frac{1}{{1 + b \times C_{0} }}$$

where *C*_*0*_ is the initial Pb(II) concentration (mg/L), *b* is the Langmuir constant (L/mg) mentioned previously, and *R*_L_ parameter is a useful indicator for estimating whether the adsorption is unfavorable (*R*_L_ > 1), linear (*R*_L_ = 1), favorable (0 < *R*_L_ < 1), or irreversible (*R*_L_ = 0).

The linearized Freundlich equation can be described as follows:

8 $${\text{In}}Q_{e} = \ln K_{F} + \frac{1}{n}\ln C_{e}$$

where *K*_*F*_ is the Freundlich constant indicative of the adsorption capacity of the adsorbent (mg·(L/mg)^1/n^), and *n* is the Freundlich exponent depicting adsorption intensity (dimensionless). In the Freundlich model, both monolayer and multiple-layer adsorptions are considered to take place during the adsorption process.

### Thermodynamics of adsorption

Thermodynamics parameters can be calculated out by using distribution coefficient, *K*_*d*_, which is dependent on temperature. The change in free energy (Δ*G*^0^), enthalpy (Δ*H*^0^) and entropy (Δ*S*^0^) related to the adsorption process can be worked out with the following three equations (Zhang et al. 2014):

9 $$\Delta G = - RT\ln K_{d}$$

10 $$K_{d} = \frac{{Q_{e} }}{{C_{e} }}$$

11 $$\ln K_{d} = \frac{{\Delta S^{0} }}{R} - \frac{{\Delta H^{0} }}{RT}$$

where *R* is the gas constant (8.314 J·mol/K), and *T* (K) is the absolute temperature. In terms of Eq. 11, Δ*H*^0^ and Δ*S*^0^ parameters can be deduced from the slope and intercept of the plot of ln*K*_*d*_ against 1/*T*.

## Results and discussion

### Characterization of CBAC

Table 1 shows the characteristics of CBAC. The BET results shown in Table 1 suggested that the activated carbon had a relatively high specific surface area (950 m^2^/g) with 84.7 % micropore and 15.3 % mesopore structure. The Boehm titration result showed that the CBAC surface was rich in carboxylic, lactone, and phenol groups.

**Table 1:** Main characteristics of CBAC

CBAC characteristics	Parameter values
Coarseness (mesh)	40–80
Strength	≥92 %
Porous characteristics	
BET specific surface area (m^2^/g)	950
Total pore volume (cm^3^/g)	0.8
Micropore (%)	84.7 %
Mesopore (%)	15.3 %
Iodine value (mg/g)	800–1050
Methylene blue value (mg/g)	120–150
Residual chlorine adsorption rate	≥85 %
Acidic oxygenous group on surface	
Carboxylic (mmol/g)	0.346
Lactonic (mmol/g)	0.253
Phenolic (mmol/g)	0.197

**Fig. 1:**
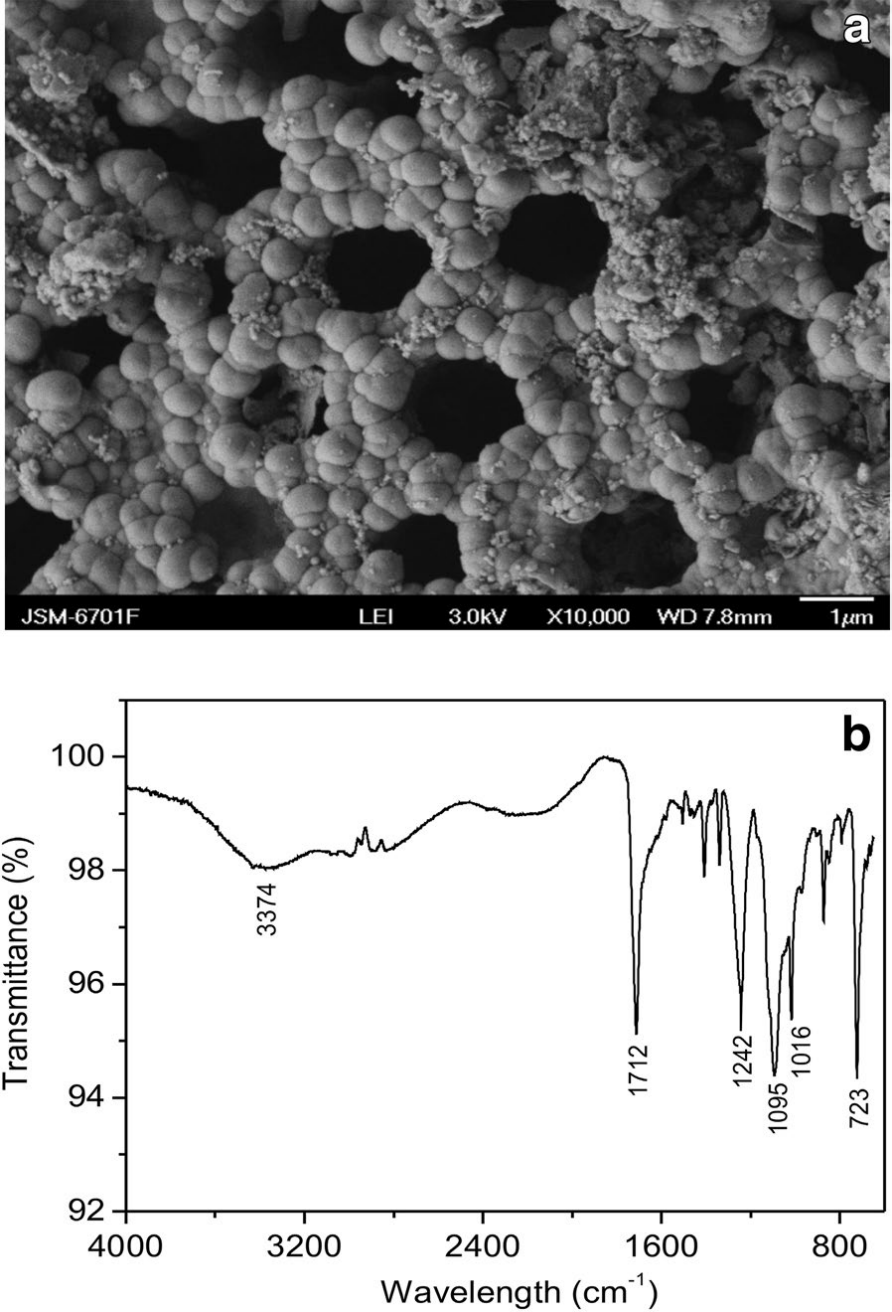
Surface characterization of CBAC: **a** SEM photo; **b** FTIR spectrum

The surface morphology of CBAC was analyzed by SEM (Fig. 1a). Obviously, the material surface has a uniform and well-developed macropore structure with the pore diameter of about 2 μm. Since the diameter of Pb(II) ion is only about 350 pm, this pore size can hold large amounts of Pb(II) ions.

Besides, the porous structure with the high total pore volume up to 0.8 cm^3^/g as well as the high specific surface area up to 950 m^2^/g could lead to the high-efficiency uptake of Pb(II).

The FTIR spectrum of CBAC is given in Fig. 1b. The broad band observed around 3374 cm^−1^ was due to O–H stretching vibration of hydroxyl, and the absorption peak at about 723 cm^−1^ was attributed to its out-of-plane bending vibration. The absorption peak at 1712 cm^−1^ could be ascribed to the C=O stretch in carboxyl groups. On the other hand, the peaks appearing at 1016, 1095 and 1242 cm^−1^ could belong to the C–OH stretching vibration. These results suggested considerable carboxyl and hydroxyl groups existed on the CBAC surface, which was in accordance with the results obtained from the above Boehm titration.

### Effect of contact time and adsorption kinetics

**Fig. 2:**
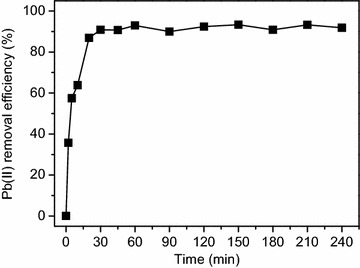
Effect of contact time on Pb(II) adsorption by CBAC (temperature: 298 K; pH: 5.0; initial Pb(II) concentration: 150 mg/L; CBAC dosage: 1.2 g/L (w/v); solution volume: 100 mL)

The effect of contact time on Pb(II) adsorption onto CBAC is shown in Fig. 2. It can be seen that the adsorption of Pb(II) onto CBAC sharply reached approximately 63 % within 10 min, then the adsorption equilibrium of Pb(II) onto CBAC is observed after 60 min. The fast adsorption rate at the initial stage might be related to a large concentration gradient between the Pb(II) in aqueous solution and that on the CBAC surface in that considerable vacant sites could be easily accessible during this period of time.

**Fig. 3:**
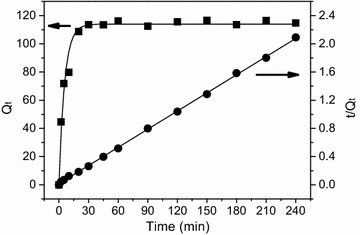
Plot of nonlinearized form of the pseudo-first-order model and linearized form of the pseudo-second-order model [temperature: 298 K; pH: 6.0; initial Pb(II) concentration: 150 mg/L; CBAC dosage: 1.2 g/L (w/v)]

To investigate the potential rate-determining step of the Pb(II) adsorption process, two common kinetics models were employed to fit the experimental data (Fig. 3). Meanwhile, the corresponding model parameters from fittings were calculated out and presented in Table 2. Obviously, *R*^2^ value of pseudo-second order model was much closer to 1.0 compared with that of pseudo-first-order model, though the *Q*_e_ values derived from the former and the latter both approached the experimental one (*Q*_exp_). This result suggested that the Pb(II) adsorption onto CBAC could be described very well by the pseudo-second-order model rather than the pseudo-first order one. Similar results have been reported on the adsorption of other heavy metals onto other carbonaceous adsorbents in the literature (Li et al. 2014; Shekinah et al. 2002; Zou et al. 2015). Therefore, it can be concluded that chemisorption may be the rate controlling step for the Pb(II) adsorption by CBAC.

**Table 2:** Kinetics parameters of the Pb(II) adsorption onto CBAC

Model	Parameter	Value
Pseudo-first-order	*k*_1_ (min^−1^)	0.1755
	*Q*_*e*_ (mg/g)	113.96
	*R* ^2^	0.9741
Pseudo-second-order	*k*_2_ (g/(mg·min))	0.0046
	*Q*_*e*_ (mg/g)	116.41
	*R* ^*2*^	0.9995
	*Q*_exp_ (mg/g)	ca. 115

### Effect of pH

**Fig. 4:**
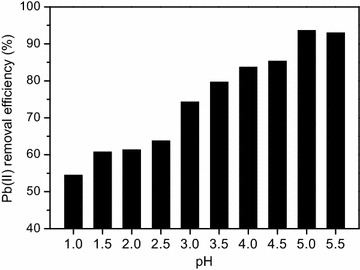
Effect of pH on Pb(II) adsorption by CBAC (temperature: 298 K; Contact time: 60 min; Pb(II) concentration: 150 mg/L; CBAC dosage: 1.2 g/L; Solution volume: 100 mL)

It is well known that solution pH is a main factor affecting the adsorption properties because of its influence on the charge state of the adsorbent surface and the degree of ionization of heavy metals in solution. The solubility product constant value (K_sp_) for Pb(OH)_2_ at 298 K is 1.42 × 10^−20^. According to the solubility product principle, the critical pH value after which the Pb(OH)_2_ precipitate began to speciate was worked out to be 5.65. In actual fact, a white precipitate could be obviously found only while the pH value elevated up to 5.94 in our practical experiment. In consideration of the formation of precipitation of metal hydroxides as pH value approached 6.0, the Pb(II) adsorption was investigated in the pH range from 1.0 to 5.5 (Fig. 4).

Obviously, the Pb(II) removal was highly pH-dependent and the optimum pH for Pb(II) adsorption was found in the pH range of 5.0–5.5 with 92.96–93.62 % of Pb(II) removed. When the pH is low, many functional groups like carboxyl and hydroxyl on the CBAC surface are protonated and existed in the positively charged species, which decreased the number of active adsorption sites (Patnukao et al. 2008). Moreover, the electrostatic repelling between the positively charged functional groups and Pb(II) could retard the binding of Pb(II) onto the surface of the adsorbents. Therefore, the adsorption of Pb(II) in acidic solution was unfavorable. While pH increased, some protonated –COOH and –OH groups are gradually deprotonated and more active adsorption sites are liberated, which could encourage the coordination of Pb(II) with these functional groups and thus enhance the removal of Pb(II) (Mouni et al. 2011). In addition, it should be noted that physisorption and chemisorption could both be involved in the Pb(II) adsorption process because CBAC took on a porous structure and had abundant hydroxyl and carboxyl groups on the surface. It could be speculated that ion exchange and coordination might serve important functions in the Pb(II) adsorption process.

### Adsorption isotherms

**Fig. 5:**
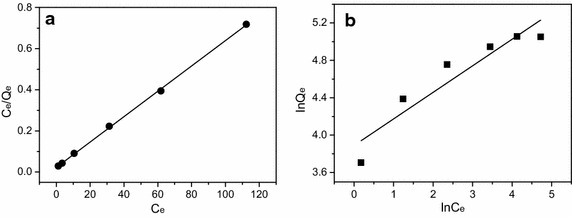
Langmuir (**a**) and Freundlich (**b**) adsorption isotherm of Pb(II) on CBAC [temperature: 298 K; contact time: 60 min; pH: 5.0; initial Pb(II) concentration: 50–300 mg/L; CBAC dosage: 1.2 g/L (w/v)]

To identify the nature of the adsorption that occurs between aqueous Pb(II) species and reactive sites in the adsorbent, equilibrium adsorption data were fitted into the Langmuir and Freundlich adsorption isotherms (Fig. 5). The isotherm constants (*Q*_max_, *b*, n, and *K*_F_) and linear correlation coefficients (*R*^2^) for Langmuir and Freundlich models are given in Table 3. Obviously, the *R*^2^ value of Langmuir model (0.9994) are nearly close to 1.0 and much greater than that of Freundlich model (0.8621). Moreover, the derived theoretical value of *Q*_max_ (162.33 mg/g) is very close to the practical adsorption capacity (156.79 mg/g). Thus, the adsorption equilibrium can be best described by the Langmuir adsorption isotherm rather than Freundlich one, implying that the Pb(II) removal may belong to monomolecular surface adsorption.

**Table 3:** Isotherm paprameters for adsorption of Pb(II) onto CBAC

Model	Parameter	Value
Langmuir	*Q*_max_ (mg/g)	162.33
	*b* (L/mg)	0.2647
	*R* ^*2*^	0.9994
	*R*_*L*_	0.0124–0.0702
Freundlich	*K*_*F*_ [(mg·(L/mg)^1/n^)]	48.8864
	*n*	3.5211
	*R* ^*2*^	0.8621

*Q*_*max*_ is a critical parameter for describing the adsorption performance of adsorbents. By comparison with other reported adsorbents, such as activated coconut shell carbon (Table 4), CBAC has a markedly higher adsorption capacity than most of those reported adsorbents, showing the great potential of CBAC for Pb(II) removal. In this study, the *R*_L_ values were calculated out in terms of Eq. 7 and listed in Table 3. The *R*_L_ values range from 0.0702 to 0.0124. This parameter (0 < R_L_ < 1) implies that the adsorption of Pb(II) onto CBAC was very favorable and CBAC is an appropriate adsorbent for the removal of Pb(II) from aqueous solution.

**Table 4:** Comparisons of adsorption capacity of various carbon materials for Pb(II)

Adsorbent	Adsorption capacity (mg/g)	pH	References
Activated carbon prepared from cotton stalk	119	4.5	Li et al. (2010)
Activated carbon from hazelnut husks	13.05	6.7	Imamoglu and Tekir (2008)
Na_2_S·HNO_3_ modified activated carbon	129.5	6	Qin et al. (2011)
Peanut shell activated carbon	35.5	2.5	Xu and Liu (2008)
Activated carbon developed from apricot stone	21.38	6.0	Mouni et al. (2011)
Carbon nanotubes	102.04	5	Kabbashi et al. (2009)
Activated carbon prepared from coconut shell	26.50	4.5	Sekar et al. (2004)
Coal-based activated carbon	162.33	5.0	This study

### Effect of temperature and adsorption thermodynamics

**Fig. 6:**
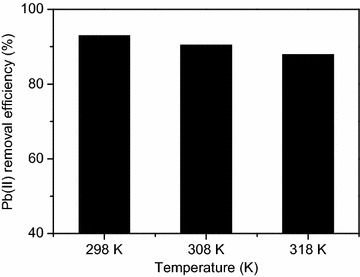
Effect of temperature on Pb(II) adsorption by CBAC (time: 60 min; pH: 5.0; initial Pb(II) concentration: 150 mg/L; CBAC dosage: 1.2 g/L; Solution volume: 100 mL)

The experiments were carried out at 298, 308 and 318 K, respectively. The Pb(II) removal efficiency changed insignificantly from 92.96 to 87.87 % when the temperature increased from 298 to 318 K (Fig. 6). This result indicated that Pb(II) adsorption by the CBAC was slightly temperature-dependent. According to Eq. 9, Δ*G*^0^ values for different temperatures were figured out, which turned out to be −5.94, −5.68 and −4.75 kJ/mol at 298, 308 and 318 K, respectively (Table 5). The negative Δ*G*^0^ values for all temperatures revealed that the adsorption of Pb(II) onto CBAC could occur spontaneously.

**Table 5:** Thermodynamics parameters for Pb(II) adsorption on CBAC

Δ*H* (kJ/mol)	Δ*S* (J mol/K)	Δ*G* (kJ/mol)		
		298 K	308 K	318 K
−23.67	−59.68	−5.94	−5.68	−4.75

**Fig. 7:**
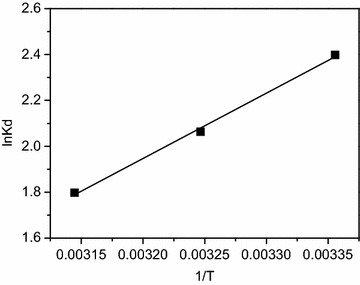
Plot of ln *K*_*d*_ ag

The Δ*H*^0^ and Δ*S*^0^ values for the adsorption process were derived from the plot of ln *K*_*d*_ versus 1/*T* (Fig. 7; Table 5). The positive value of Δ*H*^0^ (−23.67 kJ/mol) showed that the Pb(II) adsorption is an exothermic process. The positive Δ*S*^0^ value (−59.56 J·mol/K) implied that the orderliness at the solid–liquid interface increased during the adsorption process and this adsorption process was reversible. According to Gibbs–Helmholtz Equation (Δ*G*^0^ = Δ*H*^0^ − *T*Δ*S*^0^), it could be speculated that enthalpy rather than entropy was the driving force of adsorption from a perspective of thermodynamics.

## Conclusion

This study highlights the potential of using porous CBAC containing carboxyl and hydroxyl groups as an efficient adsorbent to remove Pb(II). The adsorption process was highly pH-dependent and an optimum removal with 92.96–93.62 % of Pb(II) removed was observed in the pH range of 5.0–5.5. The Pb(II) adsorption kinetics and isotherm analyses were also investigated. The results showed that the adsorption process followed pseudo-second order kinetics and proceeded at a fast rate only requiring 60 min to reach equilibrium. The adsorption isotherm model of Pb(II) onto CBAC belonged to Langmuir isotherm model and the maximum adsorption capacity was 162.33 mg/g. Thermodynamics calculations indicated that the adsorption process was favorable, spontaneous, and exothermic in nature. In conclusion, high adsorption capacity and rapid adsorption suggested that CBAC may be a promising adsorbent for removing Pb(II) from wastewater.
